# Comparing miRNAs and viroids; highly conserved molecular mechanisms for the transmission of genetic information

**DOI:** 10.3389/fncel.2014.00045

**Published:** 2014-02-20

**Authors:** James M. Hill, Walter J. Lukiw

**Affiliations:** ^1^LSU Neuroscience Center, Louisiana State University Health Sciences CenterNew Orleans, LA, USA; ^2^Department of Microbiology, Louisiana State University Health Sciences CenterNew Orleans, LA, USA; ^3^Department of Ophthalmology, Louisiana State University Health Sciences CenterNew Orleans, LA, USA; ^4^Department of Pharmacology, Louisiana State University Health Sciences CenterNew Orleans, LA, USA; ^5^Department of Neurology, Louisiana State University Health Sciences CenterNew Orleans, LA, USA

**Keywords:** micro RNAs (miRNAs), viroids, evolution, molecular speciation and complexity, miRNA-146a, genetic variation, PSTV, potato spindle tuber viroid

## Introduction

All forms of life on earth, from the most ancient *Archaebacteria* and *Lycopodiaceae* to higher eukaryotes, display phenotypes based on genetic information transfer contained within, and regulated by, hierarchic orders of RNA. While DNA is capable of storing large amounts of genetic information over long periods of time, RNA-based signaling represents a transient, highly selective, ancient, and still evolving information transfer mechanism. The information-storage capability of RNA, its solubility and the mobility of small non-coding RNA (ncRNA) species, ability to self-replicate, to catalyze chemical reactions, to direct the amino acid polymerization into peptides, and to variably regulate the expression of highly selective genetic information underscores RNA as an important, and perhaps pre-eminent, driver in the evolution of genetic complexity and information transfer (Crick, 1968; Orgel, 1968; Ambros, 2004; Sempere et al., 2004; Lukiw, 2007; Mehler and Mattick, 2007; Lukiw et al., 2008, 2012; Bartel, 2009; Kosik, 2009; Cech, 2012). This opinion paper will highlight some intriguing similarities in evolutionary ancient, molecular, and mechanistic features shared by small, non-protein coding single-stranded RNA (ssRNA) entities known as microRNAs (miRNAs), and infective, self-replicating circular ssRNA plant pathogens known as viroids. We will further highlight the intrinsic potential of miRNA and viroids to induce pathogenetic signaling in widely diverse species.

## microRNAs (miRNAs)

**Figure 1:**
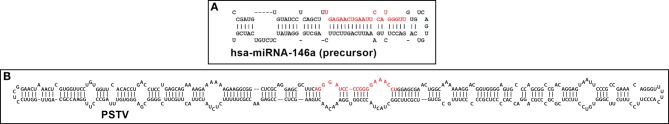
**Typical representative structures of (A) a 99 nucleotide “hairpin”-shaped precursor to a 22 nucleotide homo sapien microRNA 146a (hsa-miRNA-146a) and (B) a 359 nucleotide circular potato spindle tuber viroid (PSTV) indicate extensive intra-strand base pairing and formation of doubled stranded RNA (dsRNA), imperfect base-pairing resulting in “bulges” and stem-loop structures (Krol and Krzyzosiak, 2006; Ritchie et al., 2007; Ding, 2009; Triboulet and Gregory, 2010).** In both cases these RNA precursors are further processed by an RNase III of the family of Dicer-like proteins to generate smaller ssRNA species 18–25 nucleotides in length; these “mature” miRNA and viroid specific RNA (vsRNA) sequences are highlighted in red; note that these sizes are similar to endogenous small interfering RNA (as miRNA or vsRNA) and thus might alter the normal cultivar- and viroid-dependent gene expression in the host plant by viroids, or of mRNA in other plant and animal species, including humans (Arteaga-Vazquez et al., 2006; Krol and Krzyzosiak, 2006; Ritchie et al., 2007; Lukiw et al., 2008; Ding, 2009; Triboulet and Gregory, 2010; Hammann and Steger, 2012; Navarro et al., 2012). PSTV is the smallest known pathogen of all living species; it is interesting that both the plant and animal kingdoms have adopted similar ssRNA strategies to convey only the most essential genetic regulatory information in miRNA or vsRNA translocation and/or signal propagation, some of which may convey, in whole or part, pathological effects. Human miRNA-146a and PSTV ssRNA sequences and/or precursor structures were derived from GenBank accession NR_029701.1; GI:262205399 (http://www.ncbi.nlm.nih.gov/nuccore/NR_029701.1); miRBase (http://www.mirbase.org/cgi-bin/mirna_entry.pl?acc=MI0000477); or M36163.1; GI:333356 (http://www.ncbi.nlm.nih.gov/nuccore/M36163.1); secondary structures were predicted using online web servers such as http://mfold.rna.albany.edu/?q=mfold.

micro RNAs (miRNAs) are 18–25 nucleotide, conserved, non-coding ssRNAs that represent the smallest known carriers of highly selective genetic regulatory information in plants and animals. Mature miRNAs are typically derived from larger, double-stranded pre-miRNAs transcribed by RNA polymerase II (RNA Pol II) and processed by at least 2 RNA cleavage steps in which the activities of the RNAses Drosha and Dicer are involved, resulting in the generation of mature miRNA species (Ambros, 2004; Lukiw, 2007; Bartel, 2009; Lukiw et al., 2012). While our perceptions on the mechanism and relevance of miRNA signaling continues to evolve, it is now generally accepted that the primary mode of miRNA action is to recognize and bind to complementary RNA sequences in the 3′ un-translated region (3′-UTR) of target messenger RNAs (mRNAs) and in doing so, down-regulate their expression (Lukiw et al., 2008; Guo et al., 2010; Witkos et al., 2011; Lukiw, 2012a). While miRNAs are generally considered to be vitally essential, post-transcriptional regulators of gene expression, it is not often appreciated that these ssRNAs: (i) are very highly selected in their ribonucleotide sequence; (ii) exhibit remarkable cell and tissue specificity; (iii) represent a nucleotide signaling system that is evolutionarily ancient; (iv) are the smallest yet identified ribonucleic acid carriers of specific genetic regulatory information; (v) possess interesting viroid-like properties (see below); (vi) are the most abundant extracellular nucleic acids contained in human circulatory fluids including the cerebrospinal fluid (CSF) and blood serum; and (vii) as such may spread genetic information and gene signaling information, both homeostatic and pathogenic, amongst neighboring cells, tissues and perhaps between individual species (Arteaga-Vazquez et al., 2006; Lukiw, 2007, 2013; Yuva-Aydemir et al., 2011; Zhao et al., 2013). Rudimentary ribonucleic acid sequence analysis indicates that a “typical” 22 nucleotide ssRNA composed of the 4 different ribonucleotides (A,G,C,U) could have over 10^13^ possible sequence combinations. However, the fact that there typically only about 2 × 10^3^ different miRNAs so far identified, suggests an extremely high evolutionary selection pressure to utilize only specific ribonucleotide sequences in miRNA that will yield biologically useful miRNA–mRNA interactions (Orgel, 1968; Lukiw, 2007, 2013; Bartel, 2009). Diverse investigative methods that include miRNA array-, RNA-sequencing-, Northern dot blot-, and RT-PCR-based analysis suggest that abundant miRNAs in specialized cells such as human brain cells probably number less than ~50, and only a significantly smaller fraction of these are utilized in pathological signaling of, for example, neurological disorders (Burmistrova et al., 2007; Lukiw and Pogue, 2007; Lukiw, 2012a). Interestingly, the abundance, speciation, and complexity of these highly selected miRNAs may vary amongst different human populations in health and disease (Lukiw, 2012b, 2013). Similar to messenger RNA (mRNA), miRNAs appear to follow the same stability rules involving adenine-uridine (AU) rich elements (AREs) in their primary sequence; a higher content of AREs in miRNAs are generally correlated with short miRNA half-lives, and absence of AU or UA dinucleotides may confer miRNA stability (Chen and Shyu, 1995; Cui et al., 2005; Sethi and Lukiw, 2009). Interestingly, while mammalian brain and retinal miRNAs in particular may have in general a relatively short half-life, miRNA half-lives may be considerably extended by miRNA-binding proteins, extensive miRNA secondary and tertiary structures, circularization or interaction with RNA circles, containment within vesicles, or by combinations of these and other factors (Krol et al., 2010; Boon and Vickers, 2013; Hansen et al., 2013; Kosaka et al., 2013; Memczak et al., 2013; Perkel, 2013). In double stranded miRNA precursors, virtually all of the miRNA sequence base-pairs with complementary sequences in other parts of the same molecule to form a highly structured pre-miRNA considerably more resistant to degradation than ssRNA alone (Figure 1).

The conservation amongst specific miRNAs and ribonucleotide sequence preservation is remarkable. Using RNA-sequencing and novel genome-wide computational approaches to detect miRNAs based on nucleotide sequence structure and alignment, the miRNA-854 family has been shown to be expressed in *Arabidopsis thaliana, Caenorhabditis elegans*, *Mus musculus*, and *Homo sapiens* (Arteaga-Vazquez et al., 2006; unpublished observations). In these widely diverse species (*Arabidopsis—Homo sapiens* divergence about 1.5 billion years) miRNA-854 commonly targets an oligouridylate binding protein 1b mRNA 3′-UTR that normally encodes a member of a heterogeneous nuclear RNA binding protein family, suggesting a common origin of miRNA-854 as an ancient regulator of basal eukaryotic transcription (Wang et al., 1999; Hedges, 2002; Arteaga-Vazquez et al., 2006; Taft et al., 2010; unpublished). Moreover, secondary and/or tertiary structures may also be conserved between miRNAs, and internal stems, loops and mis-paired “bulges” commonly appear in specific positions in many pre-miRNA sequences (Figure 1) (Saetrom et al., 2006). Unique ssRNA sequences, some of which are conserved across both plant and animal species, further define and regulate the expression a relatively discrete subset of mRNAs with which they may interact, thus defining a complex, interwoven miRNA-mRNA regulatory network (Orgel, 1968; Lukiw et al., 1992; Ambros, 2004; Taganov et al., 2006; Bartel, 2009; Lukiw, 2012a,c). ssRNAs presumably also carry a signal encoded within the sequence of their RNA which may be transmitted via a unique molecular shape, molecular topology, and/or RNA charge density across the entire molecule. Intriguingly miRNAs enriched in certain foods may pass horizontally between species, carrying genetic regulatory information well outside of the cells in which they were initially generated (Alexandrov et al., 2012; Sarkies and Miska, 2013; Zhang et al., 2013).

## Viroids

The smallest known ssRNA viruses are retroviruses, such as Rous Sarcoma virus (RSV), with a 3.5 × 10^4^ nucleotide genome (Adams and Carstens, 2012; http://www.ictvonline.org/). Smaller than any known ssRNA viruses are minimalist plant pathogens known as viroids. It remains debatable whether viroids are a biological oddity, an evolutionary fossil of pre-cellular evolution, and/or a highly novel and evolving, self-perpetuating pathogenic entity (Diener, 1991, 2003; Rocheleau and Pelchat, 2006; Ding, 2009; Diermann et al., 2010; Sano et al., 2010; Wang and Ding, 2010; Navarro et al., 2012). The host range of many viroids is currently expanding, essentially as a result of a fast and continuing evolution of sequences/structures that gain new biological functions (Diener, 1991, 2003; Ding, 2009; Navarro et al., 2012). Comprising a small family of about 35 different, non-coding, un-encapsulated, autonomously infectious circular ssRNA plant pathogens ranging in size from 246 to 401 nucleotides, viroids possess the highest *in vivo* mutation rate among all known nucleic acids (Ding, 2009). Viroids are not only of evolutionary, virological and biological interest but are also of agricultural and economic concern since viroid infections compromise the yield of several important food crops worldwide (Diener, 1991, 2003; Sano et al., 2010; Wang and Ding, 2010). The first discovered viroid was the 359 nucleotide potato spindle tuber viroid (PSTV) of the viroid family *Pospiviroidae*, a circular ssRNA which primarily causes an infectious disease of potato plants (chiefly *Solanum tuberosum*) (Figure 1) (Ding, 2009; Adams and Carstens, 2012; Hammann and Steger, 2012). Viroids are transcribed using a unidirectional rolling-circle mechanism in the infected plant nuclei (for the family *Pospiviroidae;* involving stunting diseases of potato, apple, and coconut) or the chloroplast (for the viroid family *Avsunviroidae;* involving stunting diseases of avocado, eggplant, and peach). After replication, viroid precursors exit the nucleus or chloroplast via an Exportin-5 or related transport mechanism (in a fashion similar to miRNA translocation); viroids then traffic from cell to cell to establish systemic infection. Recent findings indicate that like miRNAs, viroid infection and pathogenic activity is associated with the appearance of a small viroid-specific ssRNA (vsRNA), 21–24 nucleotides in size, processed by an RNaseIII of the family of Dicer-like proteins from a double stranded RNA (dsRNA) viroid precursor (Figure 1) (Diener, 2003; Ding, 2009; Adams and Carstens, 2012; Hammann and Steger, 2012). Mature vsRNA is therefore highly analogous to miRNA in their mechanism of generation and translocation, and both have potential to not only alter the normal gene expression patterns in the host to cause disease, but also to spread these pathogenic signals to other cells and tissues via diffusion or through circulating fluids (Diener, 2003; Diermann et al., 2010; Sano et al., 2010; Wang and Ding, 2010; Adams and Carstens, 2012; Hammann and Steger, 2012). Indeed vsRNAs have sizes, structures and actions strikingly similar to both naturally occurring endogenous miRNAs, and each are known to have considerable potential to transiently or permanently alter the ssRNA-dependent gene expression patterns in the host (Diener, 2003; Ritchie et al., 2007; Ding, 2009; Hammann and Steger, 2012).

## miRNAs and viroids; extensive similarity in structure, processing, and biological function

That 21–24 nucleotide viroid-generated vsRNA are sufficient to induce diseases in plants is highly reminiscent of the finding that miRNAs, through analogous genetic mechanisms, have capabilities to contribute to disease processes in both plant and animal species (Diener, 1991, 2003; Lukiw and Pogue, 2007; Ding, 2009; Pogue et al., 2010; Hammann and Steger, 2012). Very much like miRNAs, pre-viroid ssRNAs and vsRNAs encode no proteins, have no naturally protective protein coat, do not reverse-transcribe into DNA when they replicate, and are significantly inducible by external stress or environmental factors (Diener, 2003; Ritchie et al., 2007; Adams and Carstens, 2012). Naked, infectious viroids are initially generated as a precursor dsRNA (pre-viroid) structure from which a mature ssRNA is excised by RNAse III-type nuclease activities (see Figure 1) (Ritchie et al., 2007; Lukiw, 2012b; Navarro et al., 2012; Perkel, 2013). As far as is known, miRNAs do not replicate *in vivo*; in fact they are probably physically too small to do so, and in *in vitro* experiments usually require nucleotide-linkers to effectively copy miRNAs using RT-PCR-based systems. Interestingly, circularization of pre-viroids, pri-miRNAs or miRNAs and/or the formation of complex higher order secondary and/or tertiary structures may further stabilize them (Rocheleau and Pelchat, 2006; Sethi and Lukiw, 2009; Perkel, 2013). The potential transmission and spreading of miRNA and viroid information-carrying signals from cell to cell, tissue to tissue and perhaps between species has a tremendous bearing on our understanding on the complex genetic interactions between diverse forms of life in the plant and animal kingdoms, and their symbiotic exchanges of biological information in natural environments (Hedges, 2002; Arteaga-Vazquez et al., 2006; Hammann and Steger, 2012; Sarkies and Miska, 2013).

## Concluding remarks

Intriguing similarities between the structure and function of miRNAs and viroids underscore the idea that during evolution, once nature has found and tested a successful molecular design and “*information transfer mechanism*” it is highly preserved, and this design is used repeatedly in diverse biological applications across diverse species. These biological applications range from the post-transcriptional modulation of gene expression to the potential for disease induction in both the plant and animal kingdom. Such conservation appears to lie in the molecular-genetic mechanism of miRNA and viroid ribonucleotide sequence structure and ensuing complementarity-based ssRNA-mRNA interaction. Intriguingly, ssRNA miRNA and viroid ribonucleotide sequences contain fingerprints for conservation across many diverse species, and these fingerprints represent some of the most highly conserved nucleic acid sequences known (Triboulet and Gregory, 2010; Witkos et al., 2011; Figure 1). It has recently been shown that infection of human brain cells with a high phenotypic re-activator strain of the double-stranded linear DNA herpes simplex-1 (HSV-1) induces, and then utilizes a host-specific miRNA-146a to maintain and support HSV-1 invasiveness to propagate successful infection (Figure 1; Hill et al., 2009; Lukiw et al., 2010; Ball et al., 2013). It will be interesting to ascertain if other RNA—or DNA-based “helper viruses” promote or intensify miRNA or viroid re-activities, if miRNA effects can be moderated by vsRNA or other ssRNA or dsRNA, if miRNA and viroid activities are equally affected by the presence of circular RNA (circRNA), if miRNAs are able to interact with helper viruses to modulate or amplify miRNA effects, and what potential roles other environmental factors might play in miRNA—or vsRNA-mediated gene activity and pathogenicity (Krol and Krzyzosiak, 2006; Lukiw, 2012a; Hansen et al., 2013; Memczak et al., 2013).
